# Management of Brachioradial Pruritus With Cervical Epidural Steroid Injection: A Case Report

**DOI:** 10.7759/cureus.48060

**Published:** 2023-10-31

**Authors:** Haroutiun Hamzoian, Maha Alkhayat, Michel Abdelmasih, Shehzad Choudry

**Affiliations:** 1 Neurology, Orlando Health Orlando Regional Medical Center, Orlando, USA; 2 Neurology, Orlando Regional Medical Center, Orlando, USA; 3 Pain Management, Orlando Regional Medical Center, Orlando, USA

**Keywords:** epidural steroid injection, emg-ncv, cervical mri, spinal cord, chronic itch, degenerative spine disease, pruritus, brachioradial pruritus

## Abstract

Brachioradial pruritus (BRP) is a neuropathic dysesthesia described as itching over the dorsolateral forearm. The etiology of BRP has not been fully identified but is hypothesized to be multifactorial, including sun exposure and cervical spine disease. Management of BRP is challenging, and conservative measures often fail to provide notable improvement. We present a case of a 71-year-old woman with BRP refractory to topical and oral treatment, with radiographic evidence of cervical spinal canal and neuroforaminal stenosis. Two rounds of cervical epidural steroid injections (CESI) were performed at the C6-C7 epidural space resulting in a marked improvement of symptoms. With this case report, we would like to add to the current scientific knowledge of BRP management and the potential utilization of CESIs to provide symptomatic relief to patients suffering from refractory pruritus.

## Introduction

BRP is a neuropathic dysesthesia described as itching in the upper extremities that is frequently accompanied by altered sensations such as tingling, burning, and stinging [1]. Symptoms commonly localize to dorsolateral surfaces of the arms, but they can involve the face, neck, and chest walls either unilaterally or bilaterally. Women are affected in 70% of cases, predominantly in the fifth and sixth decades, and symptoms are most common in the C5 and C6 dermatomal distribution [2].

Causes of neuropathic pruritus are unknown and often regarded as multifactorial; hypothesized mechanisms include nerve root compression syndromes, degenerative cervical spine disease [3], space-occupying lesions of the central nervous system [4], solar ultraviolet light exposure [5], inflammatory neurological diseases [6], or traumatic injury [7]. BRP is an enigmatic diagnosis causing patients to visit multiple specialties such as dermatology and neurology in search of relief. While there are no diagnostic criteria for BRP, a prompt resolution of pruritus upon ice pack placement is considered nearly pathognomonic for BRP [8]. Unfortunately, the sensation returns shortly after the removal of the ice pack and when the skin has returned to its baseline temperature. The proposed mechanism relates to peripheral nerve injury involving small-diameter, unmyelinated C-fibers of the dorsal root ganglia (DRG). Injury to the itch and pain-detecting neurons, which are anatomically indistinguishable, results in neuron gene expression causing upregulation of gastrin-releasing peptide as well as upregulation of non-neuronal cell types like astrocytes and microglia [9]. It is also important to note that there has been experimental evidence highlighting the involvement of the transient receptor potential (TRP) ion channels as a molecular sensor of chemical, thermal, and mechanical noxious stimuli in the evocation of pain and itch. Members of the TRP vanilloid subfamily such as the TRPV1, TRPV3, and TRPV4 channels have been identified as intimately involved, and experiments targeting these channels in mice have been shown to modulate pain and pathological itch [9,10].

The diagnosis of BRP is a clinical one, and additional testing is typically not recommended. However, recent studies found that many patients with BRP have chronic radiculopathy on electromyography testing and cervical spine disease on magnetic resonance imaging (MRI) [11-13]. Notable underlying pathological conditions included cervical disc protrusions, spinal canal tumors, and metastatic spread from primary cancer to the cervical region [13,14]. Nguyen et al. presented a case where a patient’s BRP resolved after a C5-C6 anterior discectomy and fusion [15]. Another study conducted by Trait et. al. presented the resolution of BRP in 10 of 14 patients after cervical spine manipulation [16]. These studies all make a case for acquiring further electrodiagnostic studies and neuroimaging to further classify how to properly treat patients suffering from this disease [11-16].

Unfortunately, there is a lack of strong evidence to suggest optimal management for BRP. Some patients may experience relief with lifestyle modification aimed at minimizing sun exposure with the addition of topical capsaicin, steroids, anesthetics, or antihistamines [17]. If failed, systemic medications can be tried, with gabapentin being reported as most efficacious at higher doses [18]. Patients with refractory cases may be referred for interventional and surgical options based on their surgical risks and patient preferences. Fluoroscopy-guided cervical epidural steroid injections (CSEIs) are an emerging treatment modality for BRP cases with cervical spine compression. With this case report, we aim to add to the generally lacking census of brachioradial pruritus patients treated with epidural steroid injections.

## Case presentation

A 71-year-old woman with a history of arthritis, migraines, anxiety, depression, hypertension, myocardial infarction with stent placement, nonalcoholic fatty liver disease, rotator cuff repair, and significant cervical spondylosis was seen at the pain management clinic for an unusual itching sensation in her forearms. She stated that she has had a severe itching sensation for multiple years intermittently lasting for over a month and resolving gradually. The itching sensation localized in her forearms bilaterally with an average of 7 in intensity on a scale of 0-10. Symptoms improved when ice packs were applied to the affected areas and worsened during times of warm weather. She was evaluated by dermatology and found no relief with physical therapy, topical betamethasone, venlafaxine, or gabapentin.

The physical exam revealed no erythema, petechia, induration, scaling, allodynia, or ulceration of her forearms. On neck manipulation, there was mild tenderness in the upper neck with minimal limitation of range of motion. Blunting of upper extremity deep tendon reflexes was also noted on examination. Electromyography, nerve conduction studies, cervical flexion-extension X-rays, and an MRI of the cervical spine were ordered for further evaluation.

Electromyography studies conducted on the bilateral upper extremity muscles showed no evidence of denervation, reinnervation, abnormal motor unit recruitment, or morphology. Nerve conduction studies of the bilateral median and ulnar motor studies were normal in distal latency, amplitude, and conduction velocities at the wrist. Bilateral radial motor studies were normal in latency, amplitude, and conduction velocities. Bilateral median and ulnar sensory responses were normal in latency and amplitude with mildly prolonged right median sensory latency. There was a significant prolongation of the right median sensory testing at the right thumb with prolonged right median F-wave latency; this was normal on the left. There was normal sensory testing in the bilateral radial sensory latency and amplitude. These findings were significant only for mild right carpal tunnel syndrome.

**Figure 1:**
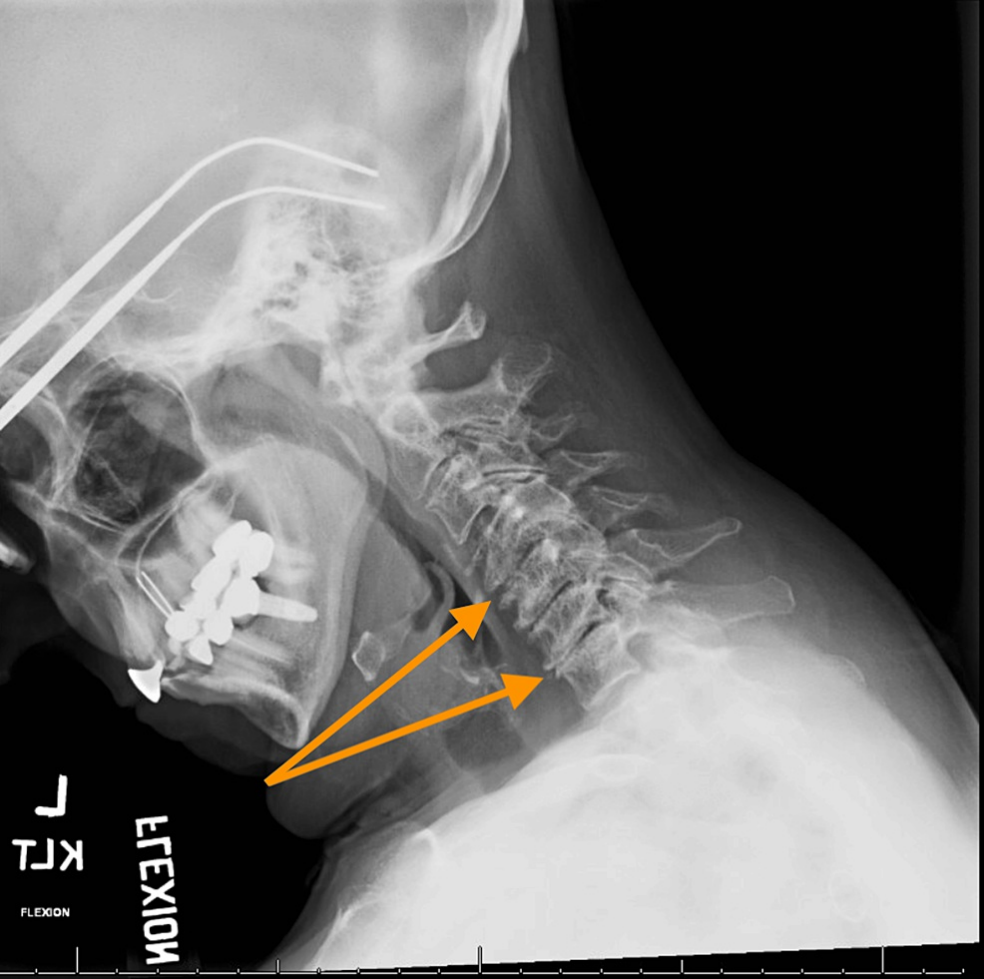
Flexion X-ray This is a left lateral flexion X-ray of our patient revealing the loss of intervertebral disc height at the cervical regions of C4–C5 and C6–C7 secondary to degenerative disc disease.

**Figure 2:**
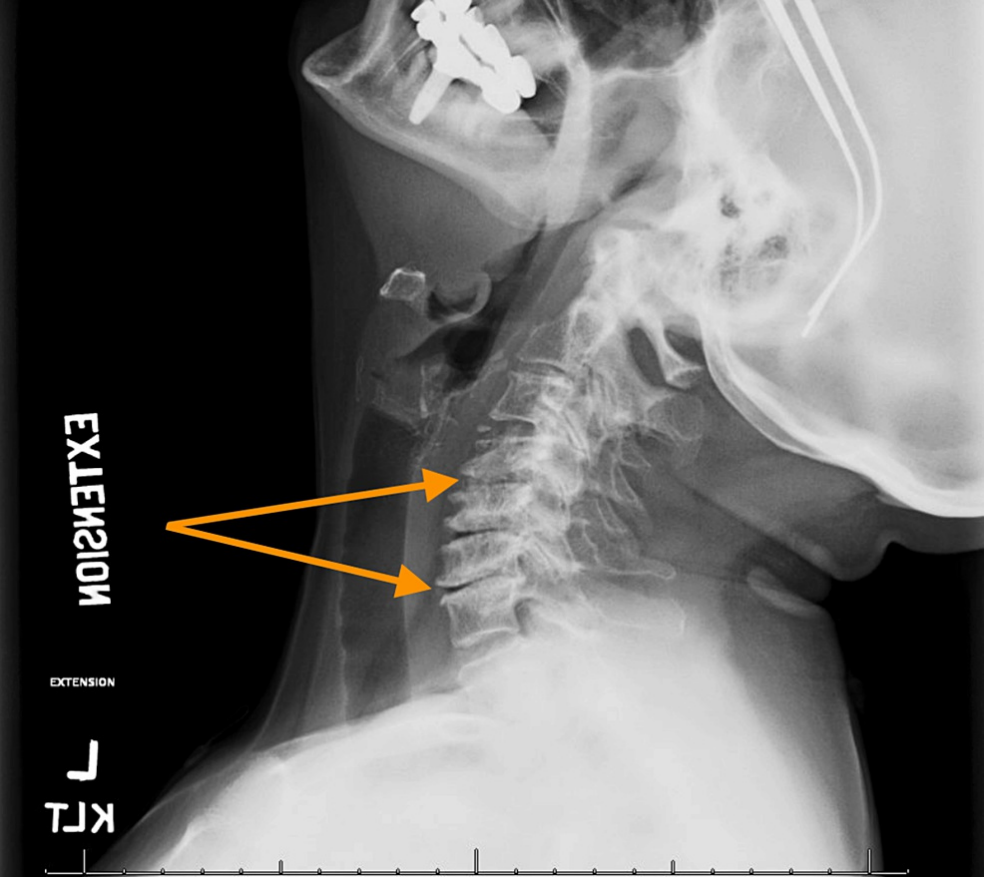
Extension X-ray This is a left lateral extension X-ray of our patient revealing the loss of intervertebral disc height at the cervical regions of C4–C5 and C6–C7 secondary to degenerative disc disease.

Imaging of the spine included cervical X-rays and MRIs. X-ray of the cervical spine with flexion and extension revealed moderate multilevel spondylosis of the cervical spine with intervertebral disc height loss between C4-C5 and C6-C7 (Figures 1, 2).

**Figure 3:**
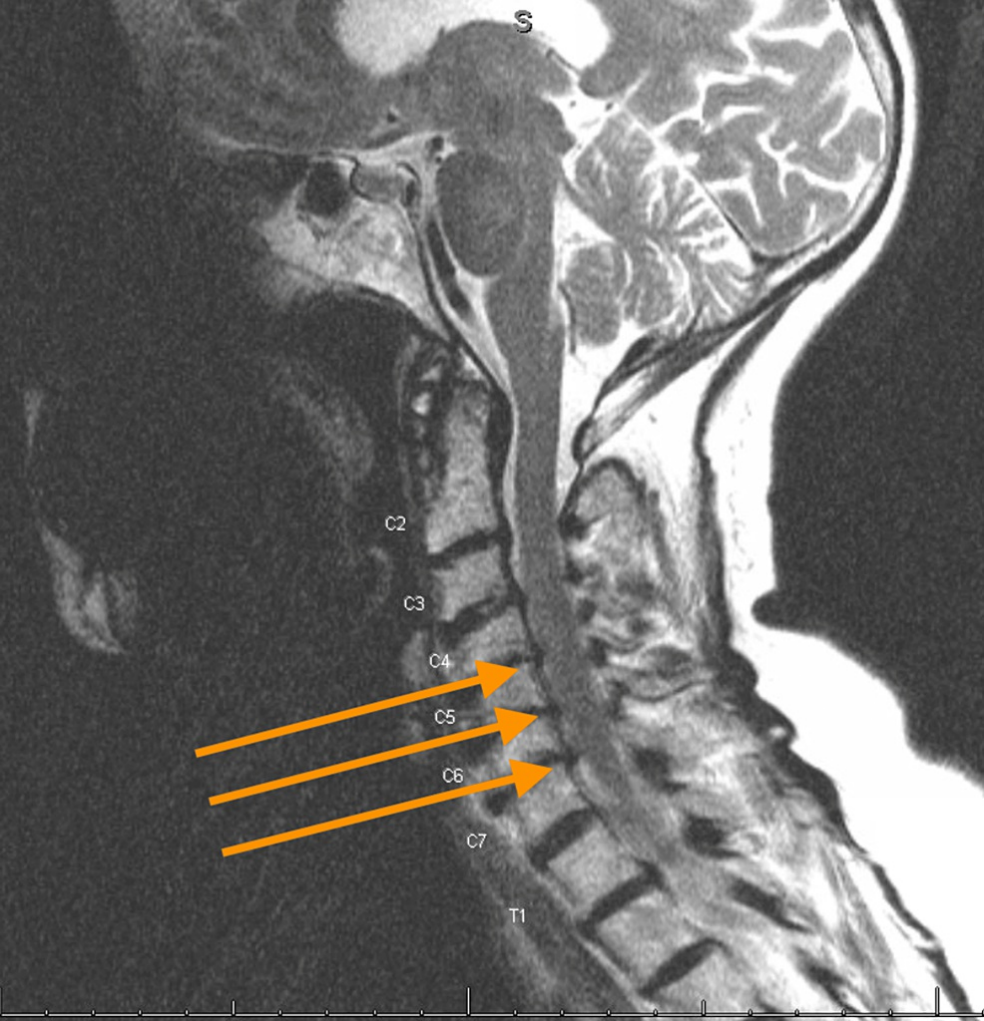
Sagittal T2 MRI of cervical spine This is a sagittal T2 MRI of the cervical spine of our patient revealing multilevel discogenic degenerative disease with central canal narrowing at the levels of C4–C5, C5–C6, and C6–C7.

**Figure 4:**
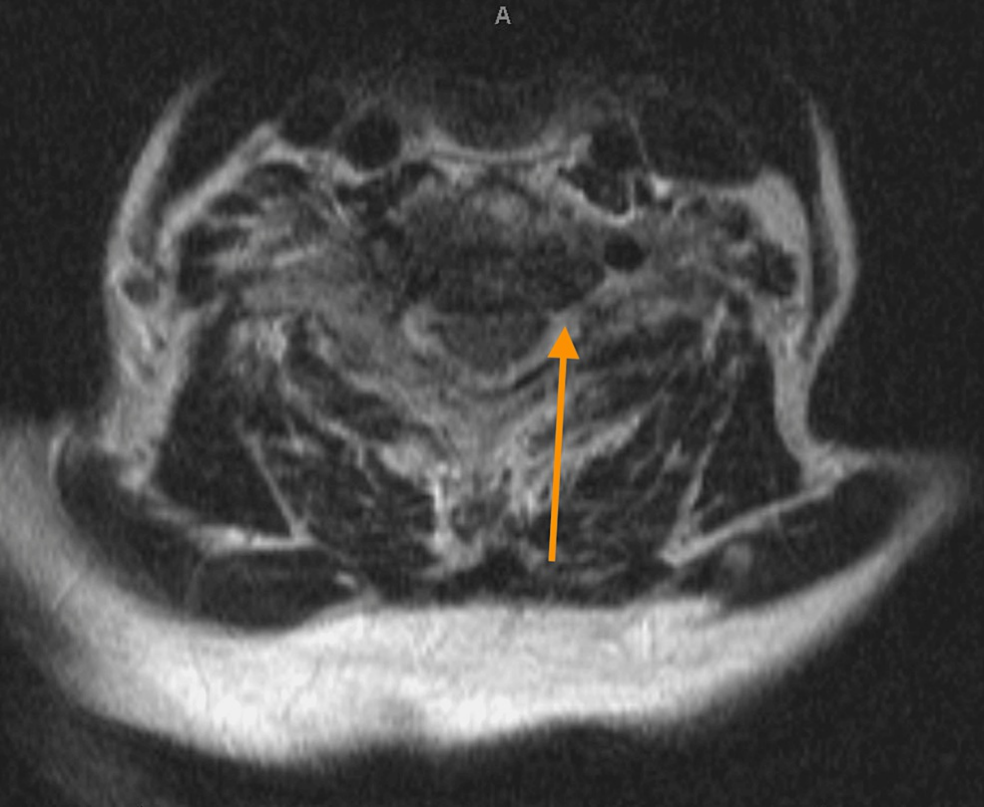
Axial T2 MRI of cervical spine This is an axial T2 MRI of the cervical spine of our patient revealing neuroforaminal narrowing at the level of C6-C7.

No spondylolisthesis or dynamic listhesis was noted. MRI of the cervical spine without contrast revealed multilevel discogenic degenerative disease with moderate disc osteophyte complexes and canal narrowing from C4-C5, C5-C6, and C6-C7 (Figure 3). Moderate bilateral neuroforaminal narrowing was notable at the C4-C5 and C5-C6 levels with worse neuroforaminal narrowing in the left at the C6-C7 level (Figure 4). No spinal cord signal was present. Paraspinal musculature and subcutaneous tissues were unremarkable with no soft tissue abnormalities noted.

**Figure 5:**
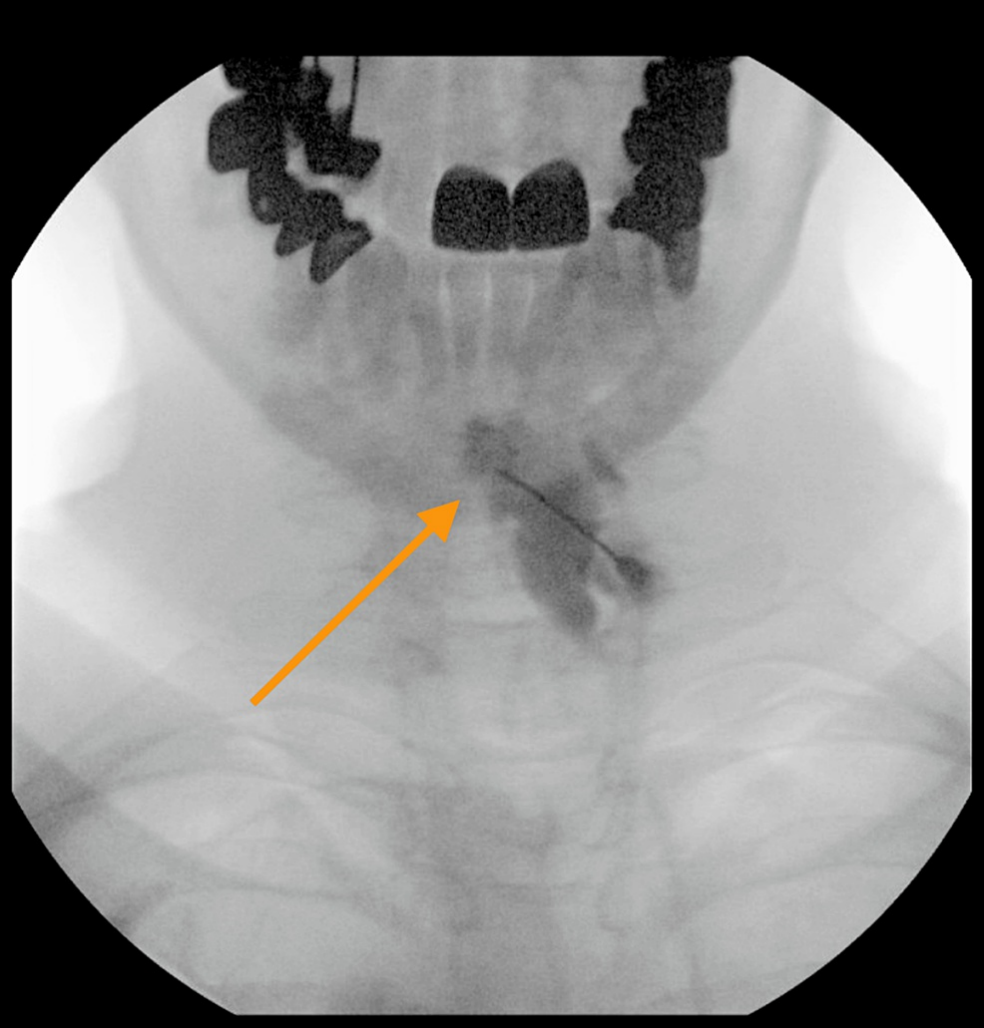
Cervical ESI #1 This is an AP fluoroscopic image of our patient's cervical spine after the omnipaque injection at the C6–C7 level for confirmatory testing before the epidural steroid solution injection. AP: Anteroposterior; ESI: Epidural steroid injection.

The patient was offered a CESI as an interventional management option to aid with her symptoms. Prior to the procedure, she was cleared by cardiology due to her moderate preoperative cardiac risk profile. The patient underwent a fluoroscopy-guided epidural steroid injection (ESI) in the C6-C7 epidural space with 40 mg Depo-Medrol and 2 ml preservative-free sodium chloride (Figure 5).

**Figure 6:**
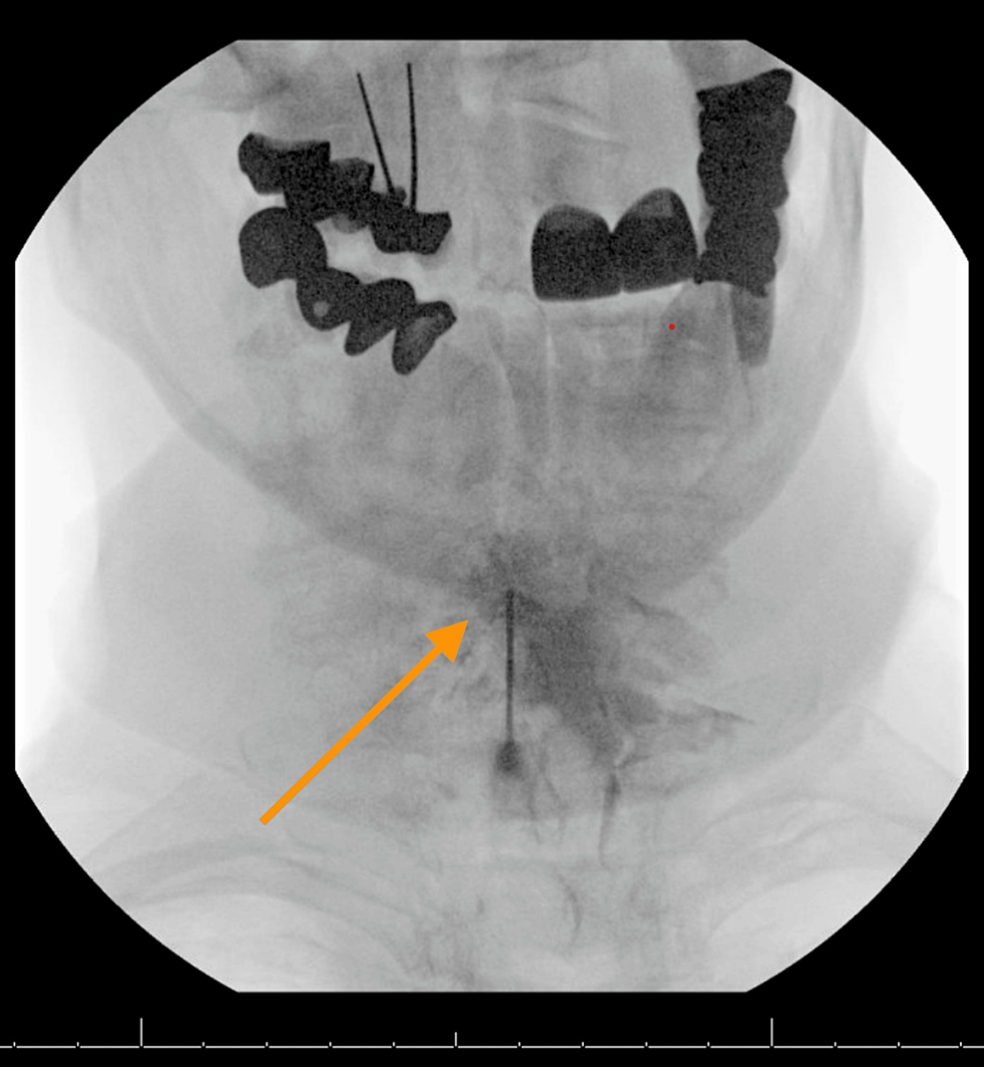
Cervical ESI #2 This is an AP fluoroscopic image of our patient's cervical spine after the omnipaque injection at the C6–C7 level for confirmatory testing before the second epidural steroid solution injection. This treatment was administered three weeks after the first ESI. AP: Anteroposterior; ESI: Epidural steroid injection.

A month after the ESI, she was seen in the clinic with an itching sensation ranked at 3/10. At that time, she endorsed a 50% symptomatic relief of the pruritus. She also stated that she felt an improvement in her neck flexion, particularly in extension and flexion. During this interval, there were no flare-ups of pruritus in the bilateral arms. Three months after her initial injection, she returned for a second injection. Another ESI was administered in the C6-C7 epidural space with 40 mg Depo-Medrol and 2 ml preservative-free sodium chloride (Figure 6). For additional support, she was advised to participate in physical therapy and prescribed lidocaine patches as adjunct therapy. The patient is currently being followed up in the clinic and was instructed to monitor for recurrence or worsening of symptoms and was offered a repeat CESI in the future, if necessary.

## Discussion

The treatment of BRP presents a challenge to healthcare professionals, primarily due to the limited efficacy of conservative measures. However, recent advancements in the field have indicated that interventions, such as the introduction of steroids into the epidural space, may offer a promising avenue for addressing BRP, particularly in patients with underlying cervical spine disease. While interventional techniques are considered relatively novel in the treatment of BRP, emerging research suggests varying degrees of pruritus relief following such interventions.

In the case of our patient, we observed a notable improvement in pruritus with a documented 50% reduction in pruritus severity, with her initial score of 7/10 dropping to 3/10. Importantly, our findings align with those reported by Weinberg et al. in their case series, where three female patients with BRP experienced favorable treatment responses following CESIs [19]. Among these patients, two achieved nearly complete resolution of their symptoms, while the third experienced substantial relief after her third CESI. Additionally, a case report by De Ridder et al. reported a middle-aged man with a three-year history of BRP and C6-C8 cervical spine pathology experiencing clinical disappearance of his pruritus after two rounds of CESIs [20]. Although we would like to concentrate on minimally invasive interventional techniques, it must be noted that the pathology of BRP, if severe enough, may also resolve following surgical intervention as previously stated. The case by Nguyen et al. highlights how severe BRP may resolve after an anterior discectomy and fusion [15]. This has not been the only documented case as Morosanu et al. also found similar results with their patient after an anterior cervical discectomy and fusion, although they mentioned that this should only be used as a last resort in patients with refractory pruritus of discogenic cause [13]. As high-quality imaging modalities become more widespread, many patients with BRP may finally find a clear reason as to why they are experiencing their symptoms. Consequently, the growing availability of interventional proceduralists and the reluctance to chronic medication use may provide BRP patients with an alternative treatment modality to improve their overall quality of life.

These case reports collectively underscore the potential therapeutic benefit of epidural injections in addressing BRP. While there is currently a lack of longitudinal studies examining the long-term effects of epidural injections in BRP patients, it is crucial to recognize that case reports, such as ours, consistently demonstrate positive outcomes in this patient population. More extensive research, like longitudinal studies, may be warranted to further explore the extent of the benefits and risks associated with interventional treatment modalities for BRP. These recent case reports and our own findings contribute to the growing body of evidence that supports the use of epidural injections as a viable treatment option for BRP patients, particularly those with cervical spine disease.

## Conclusions

In summary, there are no established criteria for selecting BRP patients that would be suitable candidates for CESIs. Our case highlights the necessity for further diagnostic imaging to understand the pathologic underpinnings of a patient’s condition. Although there are some case reports that show similar results, with therapeutic modalities ranging from interventional techniques to full surgical intervention, the total number of documented cases in the literature leaves much to be desired. Due to the positive results seen in our case report, we would like to stress the need for further evaluation and research in this field of study.

We advocate that all BRP patients should undergo cervical spine MRI to assess the potential underlying degenerative cervical pathology, particularly in cases where topical and oral treatments have proven ineffective. When conservative measures fall short, CESIs may offer a viable avenue for achieving satisfactory symptomatic relief. However, selection for this intervention should be guided by a comprehensive assessment of the patient's medical history and a thorough evaluation of operative risks. The insights gained from our case report contribute to the expanding body of knowledge on BRP management and the potential role of interventional techniques in alleviating the symptoms associated with this condition. As the scientific community continues to advance its understanding of BRP, the development of standardized criteria for CESI patient selection and a deeper exploration of its applicability will be pivotal in improving the overall care and quality of life for those afflicted by this distressing condition.
